# Standardisation of synovial biopsy analyses in rheumatic diseases: a consensus of the EULAR Synovitis and OMERACT Synovial Tissue Biopsy Groups

**DOI:** 10.1186/s13075-018-1762-1

**Published:** 2018-12-03

**Authors:** Aurélie Najm, Benoît Le Goff, Carl Orr, Rogier Thurlings, Juan D. Cañete, Frances Humby, Stefano Alivernini, Antonio Manzo, Soeren Andreas Just, Vasco C. Romão, Veit Krenn, Ulf Müller-Ladner, Olga Addimanda, Sander W. Tas, Maria Stoenoiu, Laurent Meric de Bellefon, Patrick Durez, Vibeke Strand, Mihir D. Wechalekar, Joao E. Fonseca, Bernard Lauwerys, Ursula Fearon, Douglas J. Veale, on behalf of EULAR Synovitis Study Group and OMERACT Synovial Tissue Special Interest Group

**Affiliations:** 1 Rheumatology Department, Centre Hospitalier Universitaire de Nantes, Nantes, France; 2 INSERM UMR 1238, Faculty of Biology of Nantes, Nantes, France; 3 The Centre for Arthritis and Rheumatic Diseases, Saint Vincent’s University Hospital and Dublin Academic Medical Centre, University College Dublin, Elm Park, Dublin, Ireland; 4 Institute for Molecular Life Sciences, Radboud UMC, Theodoor Craanenlaan 11, Nijmegen, 6525 GA The Netherlands; 5 Hospital Clínic de Barcelona Rheumatology Department, Arthritis Unit, Barcelona, Spain and IDIBAPS, Barcelona, Spain; 6 Centre for Experimental Medicine and Rheumatology, John Vane Science Centre, William Harvey Research Institute, Barts and the London School of Medicine and Dentistry, Queen Mary University of London, Charterhouse Square, London, EC1M 6BQ UK; 7 Division of Rheumatology, Fondazione Policlinico Universitario A. Gemelli IRCCS - Catholic University of the Sacred Heart, Rome, Italy; 8 Rheumatology and Translational Immunology Research Laboratories (LaRIT), Division of Rheumatology, IRCCS Policlinico San Matteo Foundation/University of Pavia, 27100 Pavia, Italy; 9 Department of Medicine, Svendborg Hospital, Odense University Hospital, Valdemarsgade 53, 5700 Svendborg, Denmark; 10 Rheumatology Research Unit, Instituto de Medicina Molecular, Faculdade de Medicina, Universidade de Lisboa and Rheumatology Department, Hospital de Santa Maria, Lisbon Academic Medical Centre, Lisbon, Portugal; 11 MVZ-Zentrum für Histologie, Zytologie und Molekulare Diagnostik, Trier, Germany; 12 Department of Rheumatology and Clinical Immunology, Justus-Liebig-University Giessen, Campus Kerckhoff, Giessen, Germany; 13 Medicine & Rheumatology Unit, Rizzoli Orthopaedic Institute, Bologna and Department Of Biomedical and Neuromotor Sciences, University of Bologna, 40136 Bologna, Italy; 14 Amsterdam Rheumatology and immunology Center, Department of Clinical Immunology and Rheumatology, and Laboratory for Experimental Immunology, Academic Medical Center/University of Amsterdam, Bologna, The Netherlands; 15 Department of Rheumatology, Cliniques Universitaires Saint-Luc, Institut de Recherche Expérimentale et Clinique (IREC), Université Catholique de Louvain Bruxelles, Bruxelles, Belgium; 16 Division of Immunology and Rheumatology, Stanford University, Bruxelles, CA USA; 17 Rheumatology Unit, Flinders Medical Centre and Flinders University, Adelaide, SA Australia; 18 Department of Molecular Rheumatology, Trinity Biomedical Sciences Institute, Trinity College Dublin, Dublin 2, Ireland

**Keywords:** Synovium, Standardisation, Synovial biopsy, Synovial tissue analysis

## Abstract

**Background:**

The aim of this global collaboration was to develop a consensual set of items for the analysis of synovial biopsies in clinical practice and translational research through the EULAR Synovitis Study Group (ESSG) and OMERACT Synovial Tissue Biopsy Group.

**Methods:**

Participants were consulted through a modified Delphi method. Three sequential rounds occurred over 12 months. Members were sent a written questionnaire containing items divided into two parts. Items were identified and formulated based on a scoping review. The first part of the questionnaire referred to synovial biopsies in clinical practice including five subsections, and the second part to translational research with six subsections. Every participant was asked to score each item on a 5-point Likert scale. Items with a median score above 3.5 and a ≥ 70% agreement were selected for the next round. The last round was conducted orally at EULAR in June 2017.

**Results:**

Twenty-seven participants from 19 centers were contacted by email. Twenty participants from 17 centers answered. Response rates for next rounds were 100%. For the first part relating to clinical practice, 20/44 items (45.5%) were selected. For the second part relating to translational research, 18/43 items (41.9%) were selected for the final set.

**Conclusions:**

We herein propose a consensual set of analysis items to be used for synovial biopsies conducted in clinical practice and translational research.

**Electronic supplementary material:**

The online version of this article (10.1186/s13075-018-1762-1) contains supplementary material, which is available to authorized users.

## Introduction

The synovial tissue is the target organ of many rheumatic diseases such as rheumatoid arthritis (RA). The role of synovial tissue biopsy is to provide a better understanding of disease pathophysiology, facilitate discovery of new biomarkers for diagnosis and/or prognosis and identification of new therapeutic targets [1].

Synovial biopsies (SB) have been increasingly performed over the past few years; performed for both clinical and research purposes. It is well-established that SB procedures are acceptable and well tolerated by patients, independently from the biopsy method [2, 3]. We have reported previously that SB may be useful in clinical practice [4]. In translational research, SB may be characterised and classified according to their cellular signature, the pathotype [5, 6]. Pathotypes are correlated with molecular signatures and synovial pathobiology is a promising biomarker for disease stratification and predicting the course of disease in RA [1]. Moreover, analyses of SB at both cellular and molecular levels offer a promising approach for personalised therapy in RA [1, 7]. It is highly probable that synovial biopsies will be performed more frequently in the future, and strategies including synovial tissue analysis as a central element for therapeutic decision making in RA are currently under development.

Considering this, and even though SB are performed by an increasing number of colleagues at rheumatology centres, a certain degree of heterogeneity remains in handling and analytical procedures. Harmonisation of SB handling and analytical procedures is crucial, to ensure reliability and reproducibility of the findings across different centres. Previous consensus efforts arose from EULAR Synovitis Study Group (ESSG) and Outcomes Measures for RheumAtology Clinical Trials (OMERACT) on synovial biopsy for evaluation of treatment in clinical trials [8]. These recommendations provide detailed standardised operating procedures for sample handling, however, they do not address either scoring of samples for cellular/immunological infiltrates or reporting of data concerning synovial tissue basic/translational research.

The aim of this study was to develop a process of standardisation of SB procedures. Using a modified Delphi process [9], we aimed to achieve a consensual set of items related to SB handling and analysis in both clinical practice and translational research settings. This work focused therefore on biopsy analysis and will not address biopsy retrieval methods.

## Methods

### Background work

The items were identified and formulated based on a comprehensive literature review. Item formulation was based on published articles or a centre’s experience and existing standardised operated procedures.

A task force (TF) of ESSG and OMERACT synovial tissue special interest group (SIG) members was constituted and TF members were consulted through a two-stage eDelphi process.

### Questionnaire

TF members were sent a written questionnaire containing items divided into two parts.

The first part of the questionnaire referred to clinical practice containing five subsections: biopsy sampling, biopsy handling, histological analysis, staining and immunohistochemistry (IHC), biopsy analysis and evaluation by the pathologist (Additional file 1: Table S1).

The second part referred to translational research and contained six subsections: biopsy sampling, biopsy handling, histological analysis, staining and IHC, biopsy analysis and evaluation by the pathologist, ribonucleic acid (RNA) analysis (Additional file 2: Table S2).

In the first and second rounds, every participant was asked to score each item on a 5-point Likert scale (1: strongly disagree, 2: disagree, 3: neither/neutral, 4: agree, 5: strongly agree), and comments were allowed for each item. Following each round, anonymised detailed results were communicated to participants by email.

Items with a median score above 3.5 out of 5 and a percentage of agreement above 70% were selected for the next round. Items with lower score were either suppressed of modified according to participants’ comments. Items could be added only in first round.

### First round

The first questionnaire was administered by email to the participants in June 2016.

Two email reminders were sent between June and October.

Participants were invited to comment on the items they disagreed with. Results of the first-round questionnaire were presented at the ESSG meeting in November 2016 at the American College of Rheumatology meeting in San Francisco, CA, USA.

### Second round

The second questionnaire was administered by email to participants in December 2016 (Additional file 3).

Results of the second-round questionnaire were presented at the ESSG meeting in June 2017 at the EULAR annual conference in Madrid.

### Face-to-face meeting

The face-to-face meeting occurred at the ESSG meeting in June 2017 at the EULAR annual conference in Madrid. Anonymised detailed results were sent to participants by email. This face-to-face meeting constituted a third oral round organised as follows: results were first presented and then discussed, seeking for a general agreement on the final set of items.

### Analysis

Items with a median score above 3.5 and percentage of agreement above 70% were selected for further rounds. Statistics were performed through GraphPad Prism 6.0 (GraphPad Software, San Diego, CA, USA).

## Results

### First round

Twenty-seven ESSG members from 19 centres were contacted by email. Twenty participants from 17 centres responded (response rate of 74%). Nineteen participants (95%) were rheumatologists, one participant was a pathologist (5%). Some centres (3/17) provided answers based on a consensus of the entire research team (clinician and translational scientists).

The first questionnaire contained 44 items for “Part 1 - Clinical practice”. Twenty-three items (52.3%) were selected for the second round based on their score and agreement percentage. Five items remained unchanged, 16 were modified according to participants’ comments and two were added based on participants’ suggestion.

**Fig. 1:**
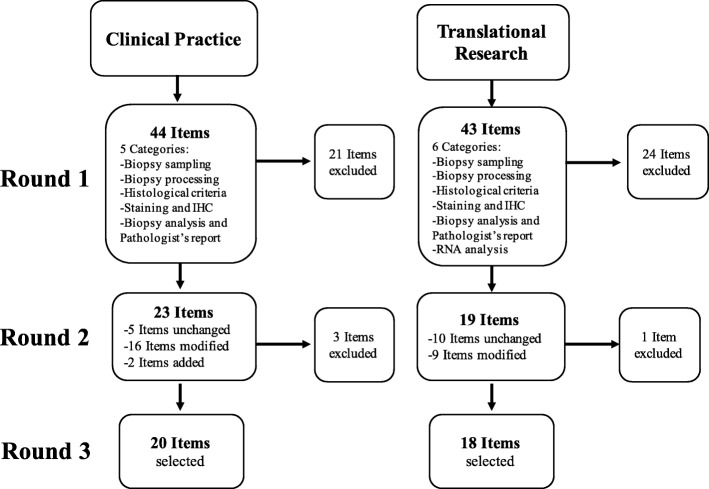
Flow chart. *IHC* immunohistochemistry, *RNA* ribonucleic acid

The first questionnaire contained 43 items for the “Part 2 - Translational research”. Nineteen items (44%) were selected for second round based on their score and agreement percentage. Ten remained unchanged, nine were modified according to participants’ comments, none was added (Fig. 1).

### Second round

For “Part 1 - Clinical practice”, 20/24 items (83.3%) were selected for the third round.

For “Part 2 - Translational research”, 18/19 items (95%) were selected for third round (Table 1).

**Table 1:** Final set of items for both clinical practice and translational research

Clinical practice	Translational research
1. Biopsy sampling - A minimum of four synovial biopsies needs to be retrieved in small joints. - Biopsies shall be retrieved in different areas of the joint, if possible. - If it is clinically relevant, bacteriological, fungal and mycobacteriological assessment should be performed. - Polymerase chain reaction analysis for RNA 16S should be performed if clinically relevant, especially if empiric antibiotic course has been started. - If it is clinically relevant, polymerase chain reaction analysis for Lyme and Whipple diseases should be performed. 2. Biopsy processing - The biopsies should spend 24 h in formalin 4%. - At least two biopsies should be formalin-fixed and paraffin-embedded. 3. Histological criteria - Synovial biopsy surface should be more than 2.5 mm^2^. - A lining layer should be seen. - Morphology of the synovial tissue should be preserved. 4. Staining and immunohistochemistry (IHC) - H&E staining should always be performed. - CD68 staining should be performed. - In particular clinically relevant cases, additional staining should be performed (CD3, CD20, CD138, CD31 or FVIII). - If performed, IHC results can be given using a semi-quantitative score. 5. Biopsies interpretation and pathologist’s report - A synovitis score should be performed, analysing: lining layer hyperplasia, inflammatory infiltrate and resident cell activation (Krenn, other). - Synovial pathotype should be described. - Presence or absence of lymphoid follicles within the membrane should be described. - Analysis can be semi-quantitative or quantitative depending on the question. - If a semi-quantitative or quantitative analysis is performed for multiple biopsies, an average score should be calculated and given for the analysis of inflammation and vascularisation. - The pathologist should mention the presence of granulomas	1. Biopsy sampling - A minimum of six synovial biopsies needs to be retrieved in large joints. - A minimum of four synovial biopsies needs to be retrieved in small joints - Biopsies shall be retrieved in different areas of the joint, if possible. 2. Biopsy processing - The biopsies should spend 24 h in formalin 4%^*^. 3. Histological criteria - Synovial biopsy surface should be more than 2.5 mm^2^. - A lining layer should be seen. - Morphology of the synovial tissue should be preserved. 4. Staining and IHC - H&E staining should always be performed. - CD68 staining should be performed. - CD3, CD19 or CD20 staining should be performed. - Additional CD 31 or FVIII, CD4, CD8, CD138 staining might be performed depending on the question. 5. Biopsies interpretation and Pathologist’s report - A synovitis score should be performed, analysing: lining layer hyperplasia, inflammatory infiltrate and resident cell activation (Krenn, other). - Lining layer hyperplasia should be scored. - Synovial pathotype should be described. - Presence or absence of lymphoid follicles within the membrane should be described. - Analysis can be semi-quantitative or quantitative depending on the question. - If a semi-quantitative or quantitative analysis is performed for a single biopsy: at least three areas of the biopsy should be assessed. 6. RNA analysis - Biopsies of one patient can be pooled for RNA extraction if needed.

^*^This item refers to those biopsies where a decision has been made to process in formalin

*CD* cluster of differentiation, *FVIII* factor VIII, *H&E* haematoxylin and eosin, *IHC* immunohistochemistry, *RNA* ribonucleic acid

### Face-to-face meeting

Results of the second round were disseminated through participating members and orally presented to the task force at the ESSG meeting in June 2017. All task force members agreed on the final set of items, as shown in Table 1.

### Items for clinical practice and translational research

#### Biopsy sampling

For the number of biopsies to be retrieved in joints, a minimum of three biopsies in large joints and of two biopsies in small joints was suggested. Nine respondents suggested to retrieve two to six biopsies, four respondents suggested to retrieve eight to 20 biopsies depending on size on the joints. For second round, to facilitate agreement, we used data previously published for translational research and suggested six biopsies to be retrieved for large joints and four biopsies for small joints.

There was some divergence in expert opinions on the need to retrieve biopsy in different areas of the joints. Some previous work suggested good correlations between the joint compartments in terms of histological analysis for cluster of differentiation (CD)68 and factor VIII (FVIII) staining [10]. Another recent work showed that a similar T cell clone exists at different regions within one joint [11]. On the other hand, it has been shown that expression of pro-inflammatory cytokines expression can vary within the same joint [12]. To facilitate agreement, we suggested to retrieve biopsies from different areas of the joint, when possible.

Many agreed that bacteriological, fungal, mycobacteriology analyses and polymerase chain reaction (PCR) for RNA 16S detection, Lyme and Whipple disease detection to be performed only when clinically relevant and not for every patient.

#### Biopsy processing

There was a strong agreement that biopsies should spend at least 24 h in formalin 4%. It was agreed that at least two biopsies should be formalin-embedded, but there was divergence in experts’ opinion regarding the necessity of snap-freezing biopsies in clinical practice.

Regarding translational research, the number of biopsies to be formalin-embedded or snap-frozen was controversial and did not reach consensus. Minimal required thickness of sections was also variating from 3 and 7 μm depending on centres and this item did not reach consensus.

#### Histological criteria

There was support for requirements regarding histological quality criteria. We suggest a biopsy size above 2.5mm^2^, a preserved lining layer as quality criteria and the overall morphology of the tissue to be preserved. Items regarding minimal requirements in the number of vessels, or percentage of stroma within the tissue required for quality check did not reach consensus.

#### Staining and immunohistochemistry

In the context of clinical practice, haematoxylin and eosin (H&E) and CD68 staining were considered to be sufficient for histopathologic analysis although some additional staining such as CD20, CD3, CD138, FVIII or CD31 could be used when clinically relevant. A semi-quantitative analysis method was felt as sufficiently accurate and less time-consuming in clinical practice, although a quantitative analysis can be performed when required by the clinical context.

Some previous work suggested that CD15 infiltrate could be strongly associated with infectious arthritis diagnosis [13]. Although some participants were strongly supportive of the use of CD15 staining when infectious arthritis was suspected, this item did not reach consensus (39.2%).

In the context of translational research, H&E, CD68, CD19 or CD20, CD3 were considered as highly relevant and required, although CD138, FVIII or CD31, CD4, or CD8 could be used depending on the scientific question. Regarding the analysis, it was argued during the meeting that previous work showed satisfactory correlation coefficients between different scoring methods, semi-quantitative or quantitative [14], and so the choice of the scoring method should depend on the question.

#### Biopsy interpretation and pathologist report

It was agreed that a synovitis score should be performed but no preference was expressed regarding the score to be used (Krenn synovitis score [15], other score). Lining layer hyperplasia scoring was recommended only for translational research purposes. Respondents overall agreed that synovial pathotype should be described although some of them emphasised that it was still not clear which features made the synovial pathotype. It was strongly felt that the presence or absence of ectopic lymphoid follicles within the membrane should be described. For clinical practice, divergent opinions were expressed regarding the need for the pathologist to suggest the likeliest diagnosis and this item finally did not reach consensus (66.7%). Some respondents argued that this should occur only when the diagnosis depended solely on the histological analysis. Conversely, mentioning the presence of granulomas was considered of importance by the panel. When multiple biopsies were analysed, a majority of respondent felt that results should be expressed as average score of the total number of biopsies analysed. For translational research, variability within the same biopsy of cell infiltrate was a matter of debate. We recommend that if a single biopsy is to be analysed, at least three different areas need to be assessed to ensure more reliability.

Although some participants were strongly supportive of assessing vascularity in clinical practice through semi-quantitative score, or translational research through the number of vessels per square millimetre or per high-power field, these items did not reach consensus (66.6% and 61.1%) (Additional file 4: Table S3).

### Items for translational research

#### RNA analysis

It was agreed by the panel that biopsies of one patient can be pooled for RNA extraction if needed.

## Discussion

Standardisation of SB analysis procedures is an important objective in the synovial tissue field and we felt it was important to combine expertise of the existing working groups: ESSG and OMERACT Synovial Tissue Biopsy Group.

In this work, we used a validated consensus method through subsequent rounds in order to achieve a consensus. The Delphi method is widely used in order to create recommendations not only in the field of rheumatology but also in other specialties [16–19].

This approach, by preserving experts’ answers anonymity, has the advantage to be less subject to peer-pressure and reduces bias.

In the current work, although there were disparities in protocols and in the number of participating experts from numerous centres, most of the items achieved consensus.

This being said, the Delphi outcome can somehow vary depending on the number of rounds [20]. We tried to address these biases by finalising the Delphi process with a face-to-face meeting to discuss and endorse the final set of recommendations.

The standardisation of SB procedures is an important objective for both clinical practice and translational research, to ensure comparability of the assessment and the reported results in multicentric studies. Extensive work has been done by the ESSG and OMERACT Synovial Tissue Biopsy Group in the past years regarding biopsy analysis and minimal requirements for clinical trials [21, 22]. Unanswered questions, however still remain. To date, despite the existence of data to support the number of biopsies to be performed in a joint or consensus on technical modalities in tissue handling [23–25], consensus is still lacking in other areas, such as minimal reporting in tissue analysis or quality requirements. Moreover, recommendations are lacking in the use of biopsies in clinical practice.

The number of centres performing synovial biopsy for clinical purposes is lower as the mini-arthroscopy technique is mostly used in the research setting in rheumatology. This implies that items related to clinical practice were formulated mostly based on expert opinion and this might represent a limitation of this work.

Providing such standardisation might, on the other hand, encourage further spreading of the biopsy procedures and generate new scientific data.

Some points that did not reach consensus in this work should be considered as specific areas for further research, for example to generate data to support decisions regarding ideal section thickness or the minimum number of area to be biopsied within the joint.

Many of the items presented in the final core set are consistent with the scientific work previously published, in terms of number of biopsies to be retrieved in large and small joints and the need for retrieval in different areas of the joint [8, 22]. Items relative to biopsy processing and histological quality requirement also displayed a high level of agreement. We observed that agreement was less frequently reached for items relating to histological analysis and immunohistochemistry. This might be explained by the fact that the immunohistochemistry performed and the scoring method used relied on the purpose of the scientific work. Indeed, two items related to vascularity scoring did not reach 70% of agreement after two rounds (agreement of 67 and 61% with median score of 3.5). This was discussed during the face-to-face meeting and it was decided that this should not prevent further use of vascularity analysis and scoring when relevant.

It is worth noting that most of the task force participants derived from the rheumatology field, as the vast majority of the centres with interest in synovial biopsies are rheumatology centres. We also invited a pathologist and a scientist with interest in synovial tissue analysis into the Task Force. The opinions did not diverge between participants regardless their background.

Despite some limitations, this consensus study provides further guidance towards standardisation of handling and analysis procedures. In future studies, different approaches could be used, in order to reduce the limitations of the Delphi approach. One approach might be to propose an approach involving two task force meetings, the first one to formulate the Delphi items and then a voting phase, finalised by a second Task Force meeting as we did.

Further step for the OMERACT Synovial Tissue Biopsy and ESSG groups will involve assessment and harmonisation of synovial tissue histological markers analysis to assess whether these can guide choices appropriate therapeutic agents, predict responses to treatment, and define a consensual set of histological items to be used for prediction of treatment responses in RA.

This work also lays the ground for further standardisation efforts in the field. The research agenda of the working groups includes a formal proposal for points to consider for minimal quality and analysis reports on synovial biopsy.

## Conclusions

In summary, we herein provide a set of consensual items on methods for synovial tissue biopsy analysis in both clinical practice and translational research, as a step towards standardisation of biopsy handling and analysis procedures across centres.

## Additional files

Additional file 1: **Table S1.** First round questionnaire for “Part 1 - Clinical practice”. (DOCX 85 kb)

Additional file 2: **Table S2.** First round questionnaire for “Part 2 - Translational research”. (DOCX 83 kb)

Additional file 3: F1. URL: Link to second-round Google form questionnaire. https://docs.google.com/forms/u/0/d/1T6CHQ7gdXqCzofgbkQL70Yg-ijyz7903MaL-78HDobk/edit?usp=forms_home&ths=true. (DOCX 14 kb)

Additional file 4: **Table S3.** Items of the second round with percentage of agreement and median score. Items in italics have not reached consensus. (DOCX 19 kb)

## Abbreviations

CD: Cluster of differentiation
ESSG: EULAR Synovitis Study Group
FVIII: Factor VIII
H&E: Haematoxylin and eosin
IHC: Immunohistochemistry
OMERACT: Outcomes Measures for RheumAtology Clinical Trials
PCR: Polymerase chain reaction
RA: Rheumatoid arthritis
RNA: Ribonucleic acid
SB: Synovial biopsies
SIG: Special interest group
TF: Task force
