# Correction to: Standardisation of synovial biopsy analyses in rheumatic diseases: a consensus of the EULAR Synovitis and OMERACT Synovial Tissue Biopsy Groups

**DOI:** 10.1186/s13075-018-1795-5

**Published:** 2018-12-19

**Authors:** Aurélie Najm, Benoît Le Goff, Carl Orr, Rogier Thurlings, Juan D. Cañete, Frances Humby, Stefano Alivernini, Antonio Manzo, Søren Andreas Just, Vasco C. Romão, Veit Krenn, Ulf Müller-Ladner, Olga Addimanda, Sander W. Tas, Maria Stoenoiu, Laurent Meric de Bellefon, Patrick Durez, Vibeke Strand, Mihir D. Wechalekar, Joao E. Fonseca, Bernard Lauwerys, Ursula Fearon, Douglas J. Veale, on behalf of EULAR Synovitis Study Group and OMERACT Synovial Tissue Special Interest Group

**Affiliations:** 1 Rheumatology Department, Centre Hospitalier Universitaire de Nantes, Nantes, France; 2 INSERM UMR 1238, Faculty of Biology of Nantes, Nantes, France; 3 The Centre for Arthritis and Rheumatic Diseases, Saint Vincent’s University Hospital and Dublin Academic Medical Centre, University College Dublin, Elm Park, Dublin, Ireland; 4 Institute for Molecular Life Sciences, Radboud UMC, Theodoor Craanenlaan 11, Nijmegen, 6525 GA The Netherlands; 5 Hospital Clínic de Barcelona Rheumatology Department, Arthritis Unit, Barcelona, Spain and IDIBAPS, Barcelona, Spain; 6 Centre for Experimental Medicine and Rheumatology, John Vane Science Centre, William Harvey Research Institute, Barts and the London School of Medicine and Dentistry, Queen Mary University of London, Charterhouse Square, London, EC1M 6BQ UK; 7 Division of Rheumatology, Fondazione Policlinico Universitario A. Gemelli IRCCS - Catholic University of the Sacred Heart, Rome, Italy; 8 Rheumatology and Translational Immunology Research Laboratories (LaRIT), Division of Rheumatology, IRCCS Policlinico San Matteo Foundation/University of Pavia, 27100 Pavia, Italy; 9 Department of Medicine, Svendborg Hospital, Odense University Hospital, Valdemarsgade 53, 5700 Svendborg, Denmark; 10 Rheumatology Research Unit, Instituto de Medicina Molecular, Faculdade de Medicina, Universidade de Lisboa and Rheumatology Department, Hospital de Santa Maria, Lisbon Academic Medical Centre, Lisbon, Portugal; 11 MVZ-Zentrum für Histologie, Zytologie und Molekulare Diagnostik, Trier, Germany; 12 Department of Rheumatology and Clinical Immunology, Justus-Liebig-University Giessen, Campus Kerckhoff, Giessen, Germany; 13 Medicine & Rheumatology Unit, Rizzoli Orthopaedic Institute, Bologna and Department of Biomedical and Neuromotor Sciences, University of Bologna, 40136 Bologna, Italy; 14 Amsterdam Rheumatology and immunology Center, Department of Clinical Immunology and Rheumatology, and Laboratory for Experimental Immunology, Academic Medical Center/University of Amsterdam, Bologna, The Netherlands; 15 Department of Rheumatology, Cliniques Universitaires Saint-Luc, Institut de Recherche Expérimentale et Clinique (IREC), Université Catholique de Louvain Bruxelles, Bruxelles, Belgium; 16 Division of Immunology and Rheumatology, Stanford University, Bruxelles, CA USA; 17 Rheumatology Unit, Flinders Medical Centre and Flinders University, Adelaide, SA Australia; 18 Department of Molecular Rheumatology, Trinity Biomedical Sciences Institute, Trinity College Dublin, Dublin 2, Ireland

**Correction to: Arthritis Res Ther (2018) 20:265**

**https://doi.org/10.1186/s13075-018-1762-1**

Following publication of the original article [1], the authors reported an error in the spelling of the ninth author’s name.

Incorrect spelling: Soeren Andreas Just.

Correct spelling: Søren Andreas Just.

The original article [1] has been updated.
