# Supplementary material for: Standardisation of synovial biopsy analyses in rheumatic diseases: a consensus of the EULAR Synovitis and OMERACT Synovial Tissue Biopsy Groups
Source: Arthritis Res Ther. 2018 Dec 3;20:265. doi: 10.1186/s13075-018-1762-1 (PMC6276172; doi:10.1186/s13075-018-1762-1)
Supplement: Supplementary file 2 — Table S2. First round questionnaire for “Part 2 - Translational research”. (DOCX 83 kb) [file 13075_2018_1762_MOESM2_ESM.docx]

**Table S2.** First round questionnaire for “Part 2- Translational Research”.
