# Supplementary material for: Standardisation of synovial biopsy analyses in rheumatic diseases: a consensus of the EULAR Synovitis and OMERACT Synovial Tissue Biopsy Groups
Source: Arthritis Res Ther. 2018 Dec 3;20:265. doi: 10.1186/s13075-018-1762-1 (PMC6276172; doi:10.1186/s13075-018-1762-1)
Supplement: Supplementary file 3 — F1. URL: Link to second-round Google form questionnaire. https://docs.google.com/forms/u/0/d/1T6CHQ7gdXqCzofgbkQL70Yg-ijyz7903MaL-78HDobk/edit?usp=forms_home&ths=true. (DOCX 14 kb) [file 13075_2018_1762_MOESM3_ESM.docx]

Additional file 4

**Supplementary Text 1 S1.**

Link to Second round Google form questionnaire.

https://docs.google.com/forms/u/0/d/1T6CHQ7gdXqCzofgbkQL70Yg-ijyz7903MaL-78HDobk/edit?usp=forms_home&ths=true
