# Supplementary material for: Standardisation of synovial biopsy analyses in rheumatic diseases: a consensus of the EULAR Synovitis and OMERACT Synovial Tissue Biopsy Groups
Source: Arthritis Res Ther. 2018 Dec 3;20:265. doi: 10.1186/s13075-018-1762-1 (PMC6276172; doi:10.1186/s13075-018-1762-1)
Supplement: Supplementary file 4 — Table S3. Items of the second round with percentage of agreement and median score. Items in italics have not reached consensus. (DOCX 19 kb) [file 13075_2018_1762_MOESM4_ESM.docx]

|  | **Clinical Practice** | | **Translational research** | |
| --- | --- | --- | --- | --- |
| **Item** | **% Agreement** | **Median score** | **% Agreement** | **Median score** |
| **1. Biopsy sampling** | | | | |
| *A minimum of 6 synovial biopsies needs to be retrieved in large joints.* | *66,70* | *4* | 77,80 | 4 |
| A minimum of 4 synovial biopsies needs to be retrieved in small joints | 72,20 | 3,5 | 77,70 | 5 |
| Biopsies shall be retrieved in different areas of the joint, if possible. | 83,40 | 5 | 83,30 | 4 |
| If it is clinically relevant, bacteriological, fungal and mycobacteriological assessment should be performed. | 94,50 | 5 | / | |
| Polymerase chain reaction analysis for RNA 16S should be performed if clinically relevant, especially if empiric antibiotic course has been started. | 77,20 | 4 |  |  |
| If it is clinically relevant, Polymerase chain reaction analysis for Lyme and Whipple diseases should be performed. | 77,80 | 5 |  |  |
| **2. Biopsy processing** | | | | |
| The biopsies should spend 24 hours in formalin 4%. | 88,90 | 5 | 72,30 | 4 |
| At least 2 biopsies should be formalin-fixed and paraffin-embedded. | 72,30 | 5 | / |  |
| **3. Histological criteria** | | | | |
| Synovial biopsy surface should be more than 2.5mm^2^. | 77,80 | 5 | 72,20 | 5 |
| A lining layer should be seen. | 88,90 | 5 | 83,30 | 5 |
| Morphology of the synovial tissue should be preserved. | 94,50 | 5 | 94,40 | 5 |
| **4. Staining and Immunohistochemistry (IHC)** | | | | |
| H&E staining should always be performed. | 100 | 5 | 100 | 5 |
| CD68 staining should be performed. | 72.3 | 4 | 94.4 | 5 |
| CD3, CD19 or CD20 staining should be performed. | / | | 94.4 | 5 |
| Additional CD 31 or FVIII, CD4, CD8, CD138 staining might be performed depending on the question. |  |  | 83.3 | 4 |
| In particular clinically relevant cases, additional staining should be performed (CD3, CD20, CD138, CD31 or FVIII). | 72.3 | 3.5 | / | |
| If performed, IHC results can be given using a semi-quantitative score. | 83.4 | 5 |  |  |
| *If infectious arthritis is suspected, CD15 staining should be performed.* | *39.2* | *3* |  |  |
| **5. Biopsies interpretation and Pathologist’s report** | | | | |
| A synovitis score should be performed, analyzing: lining layer hyperplasia, inflammatory infiltrate and resident cell activation (Krenn, other). | 88.8 | 4 | 83.4 | 4 |
| *Vascularity should be scored.* | *66.7* | *3.5* | / | |
| The absolute number of vessels per mm2 or per high power field should be assessed. | / | | 61.1 | 3.5 |
| Lining layer hyperplasia should be scored. |  |  | 83.4 | 4 |
| Synovial pathotype should be described. | 83.4 | 4 | 77.8 | 5 |
| Presence or absence of lymphoid follicles within the membrane should be described. | 83.4 | 4 | 83.4 | 5 |
| Analysis can be semi-quantitative or quantitative depending on the question. | 83.4 | 5 | 83.4 | 5 |
| If a semi-quantitative or quantitative analysis is performed for a single biopsy: at least 3 area of the biopsy should be assessed. | / | | 77.8 | 5 |
| If a semi-quantitative or quantitative analysis is performed for multiple biopsies, an average score should be calculated and given for the analysis of inflammation and vascularization. | 72.2 | 3.5 | / | |
| The pathologist should mention the presence of granulomas | 88.9 | 5 |  |  |
| *If appropriate and possible, the pathologist should mention the likeliest diagnosis.* | *66.7* | *4* |  |  |
| **6. RNA Analysis** | | | | |
| Biopsies of one patient can be pooled for RNA extraction if needed. | / | | 100 | 5 |

**Table S3.** Items of the round 2 with percentage of agreement and median score. Items in italic have not reached consensus.
